# The Dual Roles of Autophagy in Important Picornaviruses Infecting Livestock and Poultry

**DOI:** 10.3390/vetsci13060567

**Published:** 2026-06-09

**Authors:** Haibin Ma, Rongchang Liu, Ming Liao

**Affiliations:** 1 National and Regional Joint Engineering Laboratory for Medicament of Zoonosis Prevention and Control, Guangdong Provincial Key Laboratory of Zoonosis Prevention and Control, College of Veterinary Medicine, South China Agricultural University, Guangzhou 510642, China; mahaibin9111@stu.scau.edu.cn (H.M.); liurongc@foxmail.com (R.L.); 2 Fujian Provincial Key Laboratory for Avian Diseases Control and Prevention, Institute of Animal Husbandry and Veterinary Medicine, Fujian Academy of Agricultural Sciences, Fuzhou 350013, China; 3 Guangdong Engineering Technology Research Center of Biosafety and Intelligent Control for Aquatic Animals Diseases, Zhongkai University of Agriculture and Engineering, Guangzhou 510550, China

**Keywords:** selective autophagy, viral receptors, picornaviruses, immune evasion, livestock, poultry

## Abstract

Autophagy is a conserved catabolic process that degrades damaged proteins and organelles to preserve cellular homeostasis. Autophagy plays two opposing roles during viral infection. On the one hand, it can be subverted by viruses to facilitate replication and immune evasion. On the other hand, it limits viral infection by delivering viral components to lysosomes. The interaction between autophagy and important picornaviruses that infect cattle and poultry, such as SVV, EMCV, FMDV, and DHAV, is the main topic of this paper. However, comprehensive summaries focusing specifically on livestock and poultry remain limited. We summarize current research showing that these viruses evade host protection by manipulating several steps of the autophagic pathway, from initiation to lysosomal fusion, to produce replication-favorable environments. Notably, by directing the breakdown of viral capsid proteins, specific autophagy receptors such as SQSTM1/p62, NDP52, and optineurin (OPTN) serve as antiviral effectors. In response, picornaviruses have developed proteolytic strategies to inactivate these receptors, such as SVV 3C-mediated cleavage of SQSTM1 and OPTN. Moreover, different immune evasion tactics are shown by virus-specific engagement of organelle-selective autophagy, such as ER-phagy (SVV) or mitophagy (DHAV). The development of broad-spectrum antiviral treatments and autophagy-based biomarkers for livestock disease progression may benefit from an understanding of the convergent and different ways picornaviruses take advantage of the autophagic machinery.

## Simple Summary

Autophagy is a natural recycling process inside cells that removes damaged components and helps maintain health. When viruses infect livestock and poultry, autophagy can act as a defense mechanism by breaking down viral particles. However, some viruses have evolved ways to hijack this process to create a safe environment for their own replication and to evade the host’s immune system. This review focuses on four important picornaviruses that cause serious diseases in cattle and poultry: Seneca Valley virus (SVV), encephalomyocarditis virus (EMCV), foot-and-mouth disease virus (FMDV), and duck hepatitis A virus (DHAV). We summarize current knowledge on how these viruses manipulate different steps of autophagy to benefit themselves, and how host cells use selective autophagy receptors to recognize and destroy viral proteins. In response, viruses employ viral proteases to sabotage these receptors. Understanding these complex interactions may help develop new antiviral strategies and biomarkers for disease progression in livestock.

## 1. Introduction

### 1.1. Picornavirus

The family Picornaviridae comprises 147 species distributed across 63 genera. Many of these species infect livestock and poultry, producing a range of illnesses and imposing a heavy financial burden on animal husbandry (Table 1) [1]. These include the SVV, EMCV, DHAV, and FMDV, which pose major risks to farming operations and result in significant financial losses and health issues [2,3]. These viruses cause a variety of illnesses in cattle, pigs, poultry, and other hosts, from encephalomyelitis and foot-and-mouth disease to hepatitis and several systemic disorders [4]. According to recent research, autophagy has a complicated, dual role throughout these viruses’ infections. On the one hand, autophagy can inhibit viral replication by breaking down viral particles or promoting antigen presentation as a component of the host innate immune response. However, some viruses can hijack the autophagic route and exploit autophagosomal membranes to accelerate the budding of progeny virions, promote viral RNA replication, and avoid host immune surveillance [5,6].

**Figure 1:**
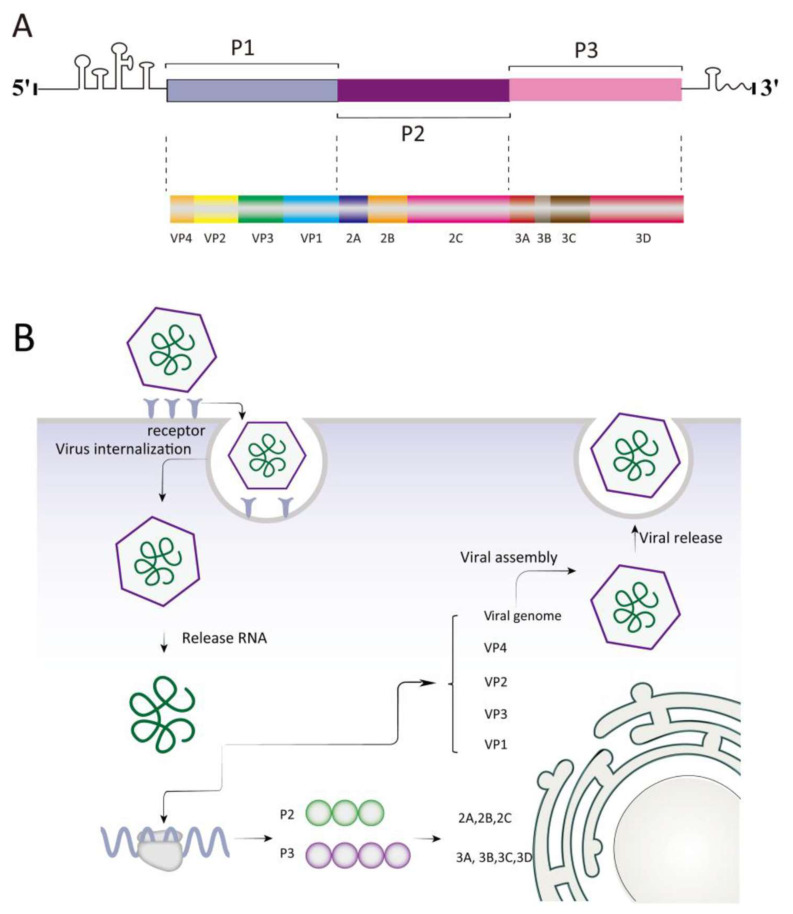
(**A**) An illustration of the picornavirus genome structure. The 5′ untranslated region (UTR) contains an internal ribosome entry site (IRES) that mediates cap-independent translation. VP1, VP2, VP3, VP4, 2A, 2B, 2C, 3A, 3B, 3C, and 3D are the products of the polyprotein’s sequential cleavage after the single ORF has been translated. A polyadenylate (polyA) tail is found at the picornavirus genome’s 3′ end. (**B**) Viral attachment, endocytosis, viral RNA release, protein translation and proteolysis, RNA replication, viral assembly, and the budding of mature virions are all phases in the picornavirus replication cycle.

The Picornaviridae family of small, non-enveloped RNA viruses has a single, continuous open reading frame (ORF) flanked by a 5′ untranslated region (UTR) and a 3′ UTR that ends in a poly(A) tail. They are between 6.7 and 10.1 kilobases in size. One feature of these viruses is the viral genome-linked protein 3B(VPg) [7]. It has a covalent bond with the positive-sense RNA strand’s 5′ end. For cap-independent translation to begin, the 5′-UTR has an internal ribosome entry site (IRES). The ORF encodes a large precursor polyprotein that is broken down by viral proteases both during and after translation to produce the capsid proteins VP0, VP1, and VP3, in addition to several nonstructural proteins like 2A, 2B, 2C, 3A, 3B, 3C, and 3D [8]. Moreover, viral RNA replication depends on the synthesis of stable precursor molecules like 3AB and 3CD [9] (Figure 1).

### 1.2. Autophagy

Autophagy is a fundamental and evolutionarily conserved catabolic system that degrades proteins and organelles to maintain cellular homeostasis [10,11]. This extensive process is classified into three main categories: macroautophagy, chaperone-mediated autophagy (CMA), and microautophagy. During microautophagy, lysosomes directly ingest cytoplasmic materials through membrane invagination [12]. The proteins that CMA particularly targets for disintegration are recognized by chaperone proteins, and they are transported to the lysosomes via a unique receptor-mediated pathway. The most prevalent mechanism for the bulk turnover of cytoplasmic components, macroautophagy, involves the creation of autophagosomes [13,14]. The autophagy pathway consists of several phases. The first sequestering compartment, the phagophore, is the first to undergo nucleation and growth [15]. The phagophore closes to form the autophagosome, a double-membraned structure that encloses the cargo [16]. The autophagosome subsequently combines with an endosome to form the acidic amphisome. In the end, the amphisome merges with a lysosome to enable the vesicular contents of the autolysosome to break down [17,18].

**Figure 2:**
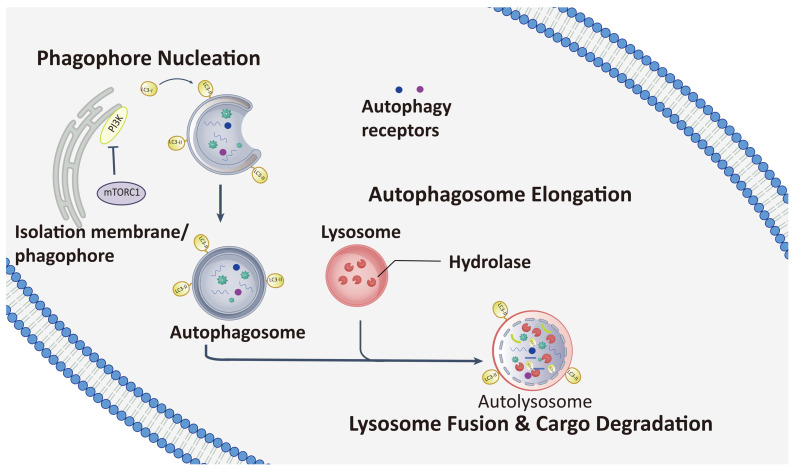
An example of the main elements and critical stages of the autophagy. Pathway include phagophore activation, autophagosome growth, lysosomal acidification, fusion, and cargo destruction. Figure 2 was created using Adobe Illustrator v29.x.

Autophagy, an essential defense mechanism in organisms, is crucial in avoiding viral infections because it sends cytoplasmic virions or viral components to lysosomes for destruction [19]. This pathway is essential for maintaining cellular homeostasis because it enables the lysosomal breakdown of misfolded proteins and the removal of damaged or malfunctioning organelles. During nutrient deprivation, it is also an essential source of energy [20,21]. Additionally, autophagy enhances pathogen elimination, inflammatory responses, and antigen presentation. Crucially, on the surface of infected cells, MHC-I and MHC-II complexes display peptides generated by the autophagic breakdown of internal pathogens, enabling immune cell identification and triggering immune responses to manage infections [22]. In order to evade the host’s immune system and facilitate their replication, research has shown that some viruses can either block or avoid autophagy, while others can control or hijack the process [23,24] (Figure 2).

### 1.3. The Mechanism and Regulation of Autophagy

#### 1.3.1. Autophagy Initiation

Autophagy initiation is primarily regulated by mTORC1, an essential inhibitor and regulatory center. Under normal circumstances, mTORC1 is active; under stressful situations, such as food scarcity or pathogen invasion, it becomes inactive, initiating autophagy [25,26]. Key signaling pathways include the RAS/RAF/MEK/ERK/mTORC1 pathway, which responds to growth factors and stress; the AMPK/mTORC1 pathway, where AMPK inhibits mTOR under energy limitation; and the PI3K/AKT/mTORC1 pathway, which integrates survival signals [27,28]. Autophagosome formation depends on the phosphorylation and activation of Class III PI3K, which leads to the production of PI3P and the recruitment of WIPI2 and DFCP1 [29]. The autophagy initiation complex, which consists of ULK1, ATG13, ATG101, and FIP200, is responsible for this. Moreover, immunological effectors like cGAS and STING1 can initiate non-canonical autophagy as a protective strategy against viral infections [30].

#### 1.3.2. Expansion and Sealing of the Autophagic Membrane

A carefully planned series of actions is involved in the autophagosome’s elongation [31]. To recruit the ATG12–ATG5–ATG16L1 complex to the phagophore assembly site, the membrane-bound protein WIPI2 first directly interacts with the ATG16L1 component [32]. Autophagosome elongation depends on this complex, which is put together by ubiquitin-like conjugation processes mediated by ATG7 (E1 enzyme) and ATG10 (E2 enzyme) [33]. The ATG12–ATG5–ATG16L1 complex then promotes the lipidation of microtubule-associated protein 1 light chain 3 beta (MAP1LC3B/LC3) by acting as an E3-like ligase. A second ubiquitin-like system—ATG7 (E1), ATG3 (E2), and the ATG12–ATG5–ATG16L1 complex itself—is involved in this lipidation [34]. Before that, pro-LC3 is cleaved by ATG4 to produce cytosolic LC3-I. Phosphatidylethanolamine (PE) and LC3-I combine to create LC3-II upon autophagy induction, which is then incorporated into the inner and outer membranes of the developing autophagosome [35]. A common indicator of autophagic activity is the level of LC3-II [19].

Cargo receptors bind on the autophagosome membrane after it has been coated with LC3-II. An LC3-interacting region (LIR) on autophagy receptors such SQSTM1/p62, NBR1, and TOLLIP allows them to bind to both LC3-II on the autophagosome membrane and particular cargo, such as ubiquitinated proteins or viral components, at the same time [36]. The cargo can be trapped in autophagosomes and then transported to lysosomes for destruction because to this bridging contact.

#### 1.3.3. Autophagic Degradation

Autophagosomes and lysosomes combine through a meticulously regulated process. The first phase is the meticulous cargo packing of the developing autophagosome. This vesicle then moves along the cytoskeleton toward the lysosome [37]. When it reaches its destination, a precise fusing event takes place, forming an autolysosome. This intricate sequence of events is coordinated by several intracellular proteins, but the SNARE superfamily, which consists of YKT6, STX17, SNAP29, VAMP3 and VAMP8, is essential [38]. They are supported by tethering proteins like the HOPS complex and the Rab GTPase family, which includes RAB7 and RAB8B. Two SNARE complexes—YKT6-SNAP29-STX7 and STX17-SNAP29-VAMP8—are necessary for the fusion process [39]. Tethering factors work as guides to bring vesicles near their target membranes for a stable and successful fusion [21]. Rubicon regulates the process and influences maturation through interactions with VPS34, ATG14L, Rab7, and UVRAG. Rab7 connects the autophagosome to the lysosome’s HOPS complex with the aid of PLEKHM1, whereas UVRAG, a part of the PI3KC3 complex, activates fusion components [40]. Within the autolysosome, lysosomal enzymes break down LC3B-II and other ingested cargo before recycling it. This degradation requires the creation of autolysosomes, which include phosphoinositides PI(3)P and PI(4)P, Rab7, and proteins such the HOPS complex and ATG14 [41]. The SNARE complex, which consists of STX17, SNAP29, and VAMP7/8, enables the final fusion phase. It delivers the contents to the lysosome so they can be destroyed. The acidic lysosomal environment must activate hydrolases in order to ensure the full breakdown of autophagic cargo [42].

### 1.4. Exploring the Interactions Between Autophagy and Picornaviruses

**Figure 3:**
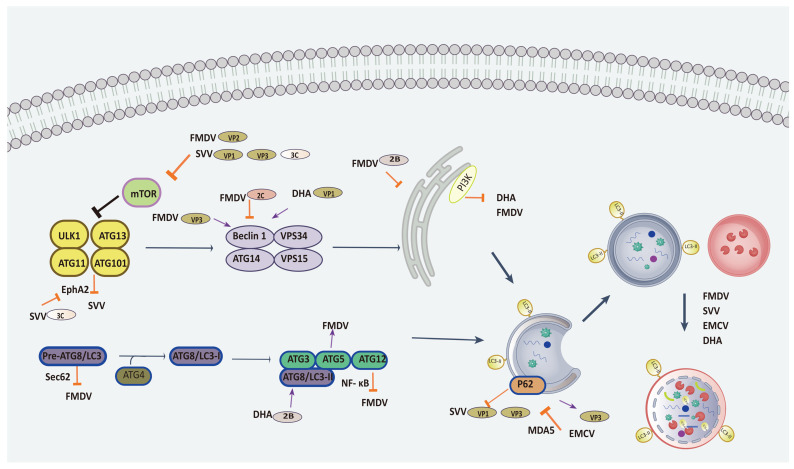
Summary of the major mechanisms by which picornavirus proteins manipulate the autophagy machinery during infection. Key phases of the autophagy pathway—initiation, elongation, closure, and fusion—involve ULK1/ATG11/ATG13/ATG101, Beclin1/VPS34/VPS15/ATG14, ATG3/ATG5/ATG12/ATG8/LC3-1 complex. Viral proteins interfere with these phases by activating mTOR (a conserved Ser/Thr kinase), interacting with autophagy-related proteins, targeting specific steps, and blocking autophagosome—lysosome fusion or lysosomal acidification, thereby subverting cellular autophagy to promote viral replication. Figure 3 was created with Adobe Illustrator v29.x.

Autophagy, a cellular degradation system, may play a protective role by limiting picornavirus replication by delivering them to autolysosomes for destruction [43]. Further study, however, demonstrates the complexity of this interaction because picornaviruses have evolved multiple strategies not only to hinder but also to hijack the autophagic machinery to promote their replication [44]. While autophagy functions as a cellular self-defense mechanism that attempts to limit infection by dismantling viral components, picornaviruses skillfully regulate different stages of autophagy to maintain their replication and survival. This connection is illustrated by the intricate interactions between viral proteins and the autophagy process. In the discussion that follows, we will examine how picornavirus proteins affect autophagy and how autophagy affects the virus in order to thoroughly examine this complex relationship. We hope this review clarifies the connection between picornaviruses and autophagy, which may aid in the future development of novel antiviral strategies (Figure 3).

This narrative review was based on literature retrieved from PubMed, Web of Science, and CNKI using keyword combinations including “autophagy”, “SVV”, “EMCV”, “FMDV”, “DHAV”, and “selective autophagy receptor”. Priority was given to original mechanistic studies and high-citation reviews published after 2010. We focus on four picornaviruses—SVV, EMCV, FMDV, and DHAV—each of which causes severe diseases in pigs, poultry, or cattle and imposes a major economic burden on livestock production. Recent studies have revealed that these viruses exploit both shared and virus-specific autophagic pathways, making autophagy a central host–pathogen battlefield during infection (Table 2).

**Table 2:** Summary of autophagy—picornavirus interactions: viral proteins, host targets, and biological outcomes.

Virus	Viral Protein(s) Involved	Host Target (Autophagy Receptor/Organelle/Pathway)	Biological Effect
FMDV	VP2, VP3, VP1 (structural); 2C, 2B, 3A, 3C (nonstructural)	HSPB1-EIF2S1-ATF4 axis; TP53-BAD-BAX; HDAC8; YTHDF2; Beclin1; G3BP1; STING1; ATG16L1; Sec62	Proviral (induces autophagy, blocks autophagosome–lysosome fusion, degrades restriction factors)
	HSPA1; Sec62; ATG5-ATG12; MCL1; HSP60	Viral 3D polymerase; IRE1α-JNK pathway; NF-κB/IRF3; mitochondrial dynamics; mitophagy	Antiviral (CMA degrades 3D; ER-phagy restores homeostasis; enhances interferon signaling)
SVV	VP1, VP3, 2B, 2C, 3C	PERK/ATF6; AKT-AMPK-MAPK-mTOR axis; STING (via FAM134B ER-phagy); cGAS; SQSTM1/p62; OPTN; EphA2	Proviral (induces bulk autophagy, cleaves SQSTM1/OPTN to evade restriction, degrades STING/cGAS)
	SQSTM1/p62; OPTN; EphA2	VP1/VP3 (cargo for selective autophagy); TBK1-IRF3 signaling	Antiviral (selective autophagy receptors directly degrade viral capsid proteins and enhance interferon response)
EMCV	2C, 3D, leader protein, VP3	TMEM39A; PERK/ATF6α; NDP52; secretory autophagy; MAVS (p62-dependent)	Proviral (induces autophagy, degrades NDP52 to evade restriction, promotes non-lytic release via secretory autophagy, suppresses MAVS signaling)
	NDP52	VP1/VP2 (cargo for autophagic degradation)	Antiviral (NDP52 directly targets capsid proteins for degradation)
DHAV-1	VP1, 2B (viroporin-like)	PI3KC3 complex; Beclin1; ER stress pathway	Proviral (activates PI3KC3-dependent autophagy, incomplete flux benefits replication)
	Mitophagy (via matrine induction); lncRNA-XR_003496198	ULK1, ULK2, EIF4EBP2; RIG-I-like receptor signaling	Antiviral (mitophagy reduces excessive interferon and pyroptosis; lncRNAs may restrict virus via autophagy regulators)

## 2. Proviral and Antiviral Functions of Autophagy During Picornavirus Infection

### 2.1. Foot-and-Mouth Disease Virus

In order to facilitate its replication, FMDV has developed a number of ways to interfere with the host autophagy system. Different autophagic pathways are activated by structural proteins like VP2, VP3, and VP1: Via HSPB1, VP2 activates the EIF2S1–ATF4 axis; VP3 initiates the TP53–BAD–BAX cascade or degrades HDAC8 via AKT–MTOR-dependent autophagy; and VP1 removes YTHDF2 to increase GTPBP4, which inhibits IRF3-mediated interferon production [45]. This proviral role is further reinforced by non-structural proteins: 2C binds Beclin1 to impede autophagosome–lysosome fusion, 2B functions as a viroporin to trigger autophagy from the endoplasmic reticulum, and 3A degrades G3BP1 via LRRC25 to reduce RIG-I-like receptor signaling [46]. Furthermore, ATG16L1 promotes early endosome trafficking, STING1 initiates PERK-dependent ER-phagy, and FMDV entry quickly induces Atg5-dependent, class III PI3K-independent autophagosome formation, all of which improve viral RNA replication [47,48].

On the other hand, autophagy can act as a host restriction mechanism to prevent FMDV. HSPA1-mediated chaperone-mediated autophagy (CMA) inhibits RNA replication by breaking down the viral RNA-dependent RNA polymerase 3D [49]. In order to inhibit viral growth, Sec62 interacts with LC3 to restore endoplasmic reticulum homeostasis and triggers the IRE1α–JNK pathway to induce autophagosome–lysosome fusion. The ATG5–ATG12 conjugation enhances interferon and ISG production by positively regulating NF-κB and IRF3 signaling; FMDV counteracts this by degrading the complex via 3Cpro. Since its deletion increases FMDV production, non-canonical autophagy that depends on ATG16L1’s WD40 domain also inhibits viral replication [50]. Furthermore, the interferon-stimulated gene MCL1 suppresses autophagy and modifies mitochondrial dynamics to prevent FMDV replication, while HSP60 regulates mitophagy to limit infection. Notably, upon infection, naturally resistant hosts like horses upregulate several autophagy genes (ATG1, ATG3, ATG9, ATG12, and ATG16L1), highlighting the autophagic pathway’s antiviral potential [51].

### 2.2. Seneca Valley Virus

As demonstrated by autophagosome formation, LC3-II accumulation, and GFP-LC3 puncta, SVV infection causes a full autophagic process in cultivated cells [52]. Pharmacological or genetic autophagy blockade significantly lowers viral yield. Mechanistically, SVV triggers autophagy through the unfolded protein response pathways PERK and ATF6, both of which are required for efficient viral replication. Additionally, the virus interacts with the AKT–AMPK–MAPK–mTOR signaling axis. The viral proteins VP1, VP3, and 3C work together to activate AKT, AMPK, and MAPKs while suppressing mTOR phosphorylation. SVV uses this route to break down important elements of the host innate immune system in addition to triggering bulk autophagy [53]. For example, SVV infection stimulates PERK/ATF6-mediated ER-phagy, which is dependent on the ER-phagy receptor FAM134B, to autophagically degrade STING, a crucial adapter for type I interferon signaling. Similar to how the 2C protein degrades cGAS via the autophagy route, the viral 2B protein enlists TOLLIP and NBR1 to target STING for autophagic turnover [54].

Additionally, selective autophagy serves as a host restriction mechanism to prevent SVV. Independent of its ubiquitin-associated domain, the autophagy receptor SQSTM1/p62 interacts with the viral capsid proteins VP1 and VP3 and directs them to phagophores for destruction [55]; overexpression of SQSTM1 inhibits the synthesis and titres of viral proteins. Similarly, optineurin (OPTN) limits viral replication by binding SVV VP1 and mediating its autophagic clearance. It also enhances TBK1–IRF3 signaling to increase type I interferon responses. To circumvent these restriction mechanisms, SVV has developed countermeasures [56]. The viral 3C protease cleaves OPTN at glutamine 513, producing N-terminal and C-terminal fragments that are unable to degrade VP1 or initiate interferon signaling, and cleaves SQSTM1 at many glutamine residues, eliminating its capacity to drive selective autophagy. Furthermore, the host protein EphA2 suppresses autophagy and inhibits SVV replication by activating the mTOR pathway; SVV 3C protease targets EphA2 for cleavage, which releases the autophagy brake and unintentionally promotes viral multiplication [57]. Therefore, SVV inhibits this by proteolytically deactivating important autophagy receptors, whereas selective autophagy is a natural antiviral defense that degrades viral components and boosts innate immunity.

### 2.3. Encephalomyocarditis Virus

Transmembrane protein 39A (TMEM39A) is upregulated by EMCV infection, which in turn stimulates viral replication in an autophagy-dependent manner. Chemical suppression of autophagy with 3-methyladenine decreases both TMEM39A expression and viral proliferation [58]. The nonstructural proteins 2C and 3D are important autophagy inducers that promote viral replication via the PERK/ATF6α axis and the endoplasmic reticulum stress pathway. Furthermore, the EMCV 2C protein evades host limitation by initiating the autophagic degradation of the autophagy adaptor NDP52, a negative regulator of viral entry and replication. Beyond intracellular replication, EMCV promotes the non-lytic budding of virions into extracellular vesicles by utilizing secretory autophagy through its leader protein. This technique probably increases viral spread while evading host immune detection. Other viral proteins, such as VP3, decrease type I interferon responses by using p62-dependent autophagy to break down the mitochondrial antiviral signaling protein (MAVS). Consistently, RNase L-induced autophagy supports late-stage EMCV replication, and pharmacological blockade of autophagy suppresses viral growth only at early time points [59].

EMCV capsid proteins VP1 and VP2 are specifically targeted by the autophagy receptor NDP52 for autophagic destruction, which prevents the virus from entering or replicating [60]. The selective pressure produced by NDP52-mediated antiviral autophagy is highlighted by the way EMCV has evolved to overcome this restriction: its 2C protein binds with NDP52 and causes its autophagic destruction via the late endosomal Rab7 and Rab9 [61,62]. Furthermore, the viral RNA sensor MDA5 is targeted for p62-dependent autophagic degradation by the E3 ubiquitin ligase RNF144B, which reduces the generation of type I interferon and hence increases EMCV replication. This second case highlights a virus-beneficial modulation of the autophagic pathway, but it also shows how the core autophagic machinery can significantly impact the course of infection when it targets viral components or critical immunological adaptors [63].

### 2.4. Duck Hepatitis a Virus

When duck embryo fibroblasts (DEFs) are infected, the virus causes endoplasmic reticulum (ER) stress, which increases LC3-II conversion and autophagosome production. Both intracellular and extracellular viral genome copies and titres are significantly reduced when autophagy is pharmacologically inhibited with chloroquine or 3MA, indicating that autophagy is functionally necessary for effective viral replication and budding. The viral structural protein VP1 directly binds PI3KC3, upregulates Beclin1 expression, and activates the PI3KC3 complex [64]. The pro-viral role of autophagy is further supported by the consistent suppression of DHAV replication by two natural polysaccharides, Chrysanthemum indicum polysaccharide (CIPS) and phosphorylated Codonopsis pilosula polysaccharide (pCPPS), which inhibit autophagosome formation and downregulate LC3-II expression. The viroporin-like 2B protein increases LC3-II levels and autophagosome numbers despite inducing incomplete autophagic flux with reduced p62 degradation, indicating that even partial autophagy may be exploited by the virus [65]. Together, these results demonstrate that DHAV actively participates in ER stress and viral protein-host interactions to initiate PI3KC3-dependent autophagy, which in turn creates a cellular milieu that facilitates viral genome replication and progeny budding [66].

Certain types of autophagy can have protective, antiviral effects against DHAV infection, despite the mostly pro-viral activity mentioned above. In vivo and in duck embryonic hepatocytes, matrine, a naturally occurring alkaloid, triggers mitophagy. This selective autophagic process reduces the excessive production of type I interferons and pyroptosis induced by DHAV-1, mitigates mitochondrial damage, and curbs hyperactivation of RLR signaling [67,68]. The autophagy inhibitor chloroquine reverses matrine’s protective properties, suggesting that mitophagy directly reduces the harmful host immunological response and pathology linked to DHAV infection. Moreover, differently expressed long noncoding RNAs (lncRNAs) have been found using transcriptome profiling of infected DEFs. Among them, lncRNA-XR_003496198 inhibits DHAV replication; important autophagy regulators like ULK1, ULK2, and EIF4EBP2 are among its probable target genes, suggesting a possible lncRNA-mediated restriction mechanism that might implicate autophagic pathways [69,70]. Therefore, the host can use mitophagy and lncRNA-regulated mechanisms to combat infection and reduce immunopathology, even while DHAV frequently coopts traditional autophagy for its life cycle.

## 3. Discussion

Despite their distinct hosts and pathologies, FMDV, SVV, EMCV and DHAV have converged on a shared strategy: inducing early autophagic flux while blocking late degradation [18]. All four viruses enhance LC3-II lipidation and activate the PI3KC3–Beclin1 axis, but they also impede autophagosome–lysosome fusion or lysosomal acidification, a phenomenon known as “arrested autophagy” [71]. FMDV 2C and DHAV 2B, which both increase autophagosome numbers without effective cargo turnover, serve as the best examples of this [72]. Similar to the replication organelles produced by other positive-strand RNA viruses, the result is a membrane-rich, degradative-compromised compartment that acts as a scaffold for viral RNA replication. This convergent manipulation implies that picornaviruses have evolved to separate autophagy’s membrane-generating function from its destructive potential; this idea may apply to other Picornaviridae family members [73].

One important finding from recent research is that the main antiviral defense against picornaviruses is made up of selective autophagy receptors rather than bulk macroautophagy. Viral capsid proteins (such as SVV VP1/VP3 and EMCV VP1/VP2) are directly bound by SQSTM1/p62, NDP52, and OPTN, which then target them for autophagic destruction. Importantly, picornaviruses have developed defense mechanisms that selectively block these receptors [74,75]. SQSTM1 and OPTN are cleaved at numerous glutamine residues by SVV 3C protease, while NDP52 is autophagically degraded by EMCV 2C [53]. Conversely, viruses rarely target LC3 or core ATG proteins, suggesting that they fine-tune rather than completely eliminate the route to prevent total loss of autophagic membrane supplies [76]. This receptor-centric perspective redefines the autophagy–picornavirus interface: the integrity of receptor-mediated cargo capture is more important for the outcome of infection than autophagic flux in and of itself [77].

Picornaviruses influence immune responses by differentially engaging ER-phagy and mitophagy in addition to traditional macroautophagy. SVV blunts type I interferon responses by actively subverting ER-phagy via the FAM134B receptor to destroy STING [78]. This mechanism has not yet been documented for other picornaviruses. On the other hand, mitophagy suppresses DHAV infection; matrine, a naturally occurring substance, restores mitophagy to minimize mitochondrial damage and excessive RIG-I signaling, indicating a protective role for selective organelle clearance [79]. In contrast, EMCV uses its leader protein to initiate secretory autophagy, which releases virions inside extracellular vesicles—a non-lytic egress pathway that could aid in immune evasion. Each virus’s distinct susceptibility to particular innate pathways is probably reflected in these different tactics [80]. It is still unclear why certain picornaviruses target STING (SVV), while others target MAVS (EMCV) or completely avoid organelle-specific autophagy (FMDV). Replication kinetics and tissue tropism may play a role [81].

Treatment options are made possible by the predominance of proviral autophagy during active infection. All four viruses’ replication is consistently reduced by pharmacological autophagy inhibitors (3 methyladenine, chloroquine), although their clinical application in livestock is restricted due to toxicity and off-target effects [82,83]. Stabilizing selective autophagy receptors—for instance, by creating small compounds that prevent 3C-mediated cleavage of SQSTM1 or OPTN, so preserving antiviral cargo degradation—would be a more sophisticated strategy [84,85]. On the other hand, immunopathology might be lessened by increasing mitophagy (as with matrine against DHAV) without significantly reducing autophagy. There are still a number of important unanswered questions: (i) Why do certain picornaviruses prevent autophagosome–lysosome fusion whereas others do not? (ii) Is it possible to use the ratio of cleaved to full-length SQSTM1 in tissue samples as a biomarker of the stage of viral replication? (iii) Do naturally resistant hosts show constitutively increased expression of specific autophagy receptors, such as horses for FMDV? Quantitative, spatiotemporally resolved in vivo models that go beyond LC3-II immunoblots will be necessary to address these problems. In the end, a more thorough knowledge of the mechanisms by which picornaviruses control selective autophagy may provide broad-spectrum antiviral targets that can be used against a variety of livestock infections.

## Figures and Tables

**Table 1:** Summary of picornavirus genera and species infecting domestic livestock and poultry.

Genus	Representative Virus	Primary Host	Disease/Clinical Features
Anativirus	Anativirus	Ducks	Pathogenicity unclear
Aphthovirus	Foot-and-mouth disease virus	Cattle, pigs, sheep, goats	Vesicles on mouth and feet; highly contagious
Avihepatovirus	Duck hepatitis A virus	Ducks	Acute hepatitis in ducklings
Avihepatovirus	Novel duck picornavirus	Ducks	high homology with Avihepatovirus
Avisivirus	Avisivirus	Chickens	Associated with enteric disease
Boosepivirus	Boosepivirus	Cattle, sheep, goats	Potential gastroenteric pathogen; emerging
Cardiovirus	Encephalomyocarditis virus	Pigs, rodents, cattle	Myocarditis, encephalitis; high mortality in young pigs
Enterovirus	Swine vesicular disease virus	Pigs	Vesicular lesions on feet and mouth
Enterovirus	Bovine enterovirus	Cattle	Usually subclinical; mild enteric/respiratory signs
Gallivirus	Gallivirus	Chickens	Associated with enteric disease
Kobuvirus	Porcine kobuvirus	Pigs	Associated with enteric health; unclear pathogenicity
Kobuvirus	Bovine kobuvirus	Cattle	Associated with diarrhea
Kobuvirus	Ovine kobuvirus	Sheep	Detected in healthy sheep (2025 study)
Megrivirus	Megrivirus	Chickens, turkeys	Associated with enteric disease
Sapelovirus	Porcine sapelovirus	Pigs	Diarrhea, pneumonia, reproductive disorders; emerging
Senecavirus	Senecavirus A	Pigs	Vesicular disease (similar to FMD); emerging pathogen
Sicinivirus	Sicinivirus	Chickens	Associated with enteric disease
Teschovirus	Porcine teschovirus	Pigs	Encephalomyelitis, diarrhea, reproductive disorders
Tremovirus	Avian encephalomyelitis virus	Chickens	Neurological disease in young chicks
Unassigned/novel genus	Suluvirus	Cattle	Associated with calf diarrhea

Note: Genera are listed alphabetically.

## Data Availability

No new data were created or analyzed in this study. Data sharing is not applicable to this article.

## Abbreviations

The following abbreviations are used in this manuscript:

AMPK	AMP-activated protein kinase
ATF6	Activating transcription factor 6
ATG	Autophagy-related protein
CMA	Chaperone-mediated autophagy
DEFs	Duck embryo fibroblasts
DFCP1	Double FYVE-containing protein 1
DHAV	Duck hepatitis A virus
EIF2S1	Eukaryotic translation initiation factor 2 subunit 1
EMCV	Encephalomyocarditis virus
ER	Endoplasmic reticulum
FAM134B	Family with sequence similarity 134 member B
FMDV	Foot-and-mouth disease virus
G3BP1	Ras-GTPase-activating protein-binding protein 1
GFP	Green fluorescent protein
HDAC8	Histone deacetylase 8
HOPS	Homotypic fusion and protein sorting complex
HSP60	Heat shock protein 60
HSPA1	Heat shock protein family A member 1
HSPB1	Heat shock protein family B member 1
IRES	Internal ribosome entry site
IRF3	Interferon regulatory factor 3
ISG	Interferon-stimulated gene
LC3	Microtubule-associated protein 1 light chain 3
LIR	LC3-interacting region
lncRNA	Long non-coding RNA
LRRC25	Leucine-rich repeat-containing protein 25
MAPK	Mitogen-activated protein kinase
MAVS	Mitochondrial antiviral signaling protein
MCL1	Myeloid cell leukemia 1
MDA5	Melanoma differentiation-associated protein 5
mTORC1	Mechanistic target of rapamycin kinase complex 1
NBR1	Neighbor of BRCA1 gene 1
NDP52	Nuclear dot protein 52
NF-κB	Nuclear factor kappa B
OPTN	Optineurin
ORF	Open reading frame
PERK	Protein kinase R-like endoplasmic reticulum kinase
PI3K	Phosphoinositide 3-kinase
PI3KC3	Class III PI3K
PI3P	Phosphatidylinositol 3-phosphate
RAB	Ras-related protein in brain
RIG-I	Retinoic acid-inducible gene I
RLR	RIG-I-like receptor
RNF144B	Ring finger protein 144B
SNAP29	Synaptosome-associated protein 29
SNARE	Soluble N-ethylmaleimide-sensitive factor attachment protein receptor
STING1	Stimulator of interferon response cGAMP interactor 1
STX17	Syntaxin 17
SVV	Seneca Valley virus
TBK1	TANK-binding kinase 1
TMEM39A	Transmembrane protein 39A
TOLLIP	Toll-interacting protein
TP53	Tumor protein p53
ULK1	Unc-51-like autophagy-activating kinase 1
UVRAG	UV radiation resistance-associated gene protein
VPg	Viral genome-linked protein
WIPI2	WD repeat domain phosphoinositide-interacting protein 2
YKT6	Synaptobrevin homolog YKT6
YTHDF2	YTH domain-containing family protein 2
3MA	3-methyladenine
